# miR-181d/RBP2/NF-κB p65 Feedback Regulation Promotes Chronic Myeloid Leukemia Blast Crisis

**DOI:** 10.3389/fonc.2021.654411

**Published:** 2021-03-25

**Authors:** Minran Zhou, Xiaolin Yin, Lixin Zheng, Yue Fu, Yue Wang, Zelong Cui, Zhenxing Gao, Xiaoming Wang, Tao Huang, Jihui Jia, Chunyan Chen

**Affiliations:** ^1^ Department of Hematology, Qilu Hospital of Shandong University, Jinan, China; ^2^ Department of Microbiology/Key Laboratory for Experimental Teratology of the Chinese Ministry of Education, School of Basic Medical Science, Shandong University, Jinan, China; ^3^ Department of Pediatrics, Qilu Hospital of Shandong University, Jinan, China

**Keywords:** miR-181d, RBP2, p65, CML blast crisis, cell proliferation

## Abstract

**Background:**

Chronic myeloid leukemia (CML) is a malignant clonal proliferative disease. Once it progresses into the phase of blast crisis (CML-BP), the curative effect is poor, and the fatality rate is extremely high. Therefore, it is urgent to explore the molecular mechanisms of blast crisis and identify new therapeutic targets.

**Methods:**

The expression levels of miR-181d, RBP2 and NF-κB p65 were assessed in 42 newly diagnosed CML-CP patients and 15 CML-BP patients. Quantitative real-time PCR, Western blots, and cell proliferation assay were used to characterize the changes induced by overexpression or inhibition of miR-181d, RBP2 or p65. Luciferase reporter assay and ChIP assay was conducted to establish functional association between miR-181d, RBP2 and p65. Inhibition of miR-181d expression and its consequences in tumor growth was demonstrated *in vivo* models.

**Results:**

We found that miR-181d was overexpressed in CML-BP, which promoted leukemia cell proliferation. Histone demethylase RBP2 was identified as a direct target of miR-181d which downregulated RBP2 expression. Moreover, RBP2 inhibited transcriptional expression of NF-κB subunit, p65 by binding to its promoter and demethylating the tri/dimethylated H3K4 region in the p65 promoter locus. In turn, p65 directly bound to miR-181d promoter and upregulated its expression. Therefore, RBP2 inhibition resulting from miR-181d overexpression led to p65 upregulation which further forwarded miR-181d expression. This miR-181d/RBP2/p65 feedback regulation caused sustained NF-κB activation, which contributed to the development of CML-BP.

**Conclusions:**

Taken together, the miR-181d/RBP2/p65 feedback regulation promoted CML-BP and miR-181d may serve as a potential therapeutic target of CML-BP.

## Introduction

Chronic myeloid leukemia (CML) is a malignant myeloproliferative disease that is originated from hematopoietic stem cells and characterized by the *BCR-ABL1* fusion gene (1). The natural course of the disease includes a chronic phase (CP), an accelerated phase (AP) and a blast phase (BP). In the chronic phase of CML (CML-CP), the leukemia cells still retain a certain ability to differentiate into mature cells; while in blast phase of CML (CML-BP), a large number of invasive primitive and immature cells accumulate (2). At present, CML-CP patients have a good response to tyrosine kinase inhibitors (TKIs), and most of them survive for a long time. However, some patients are still insensitive to TKIs or develop drug resistance, leading to an accelerated phase and/or blast phase. Once they progress into CML-BP, the curative effect is poor, and the fatality rate is extremely high. Besides, the etiology of CML malignant transformation is complex and highly heterogeneous (3). Therefore, it is urgent to uncover the underlying mechanisms of CML-BP.

Epigenetics mean that the gene expression undergoes heritable changes without changes in the DNA sequence (4). Epigenetic modifications include DNA methylation, histone modification and non-coding RNA regulation, which affect cell proliferation, differentiation and apoptosis (5, 6). MicroRNAs (miRNAs) are a class of small non-coding RNAs, which negatively or positively regulate gene expression, by directly binding to the 3′untranslated regions (3′UTR) mRNAs of the target genes (7). They are also vital players in the development of leukemia. A recent study showed that miR-150 expression, was downregulated in the transition period between the CML chronic phase and the blast crisis (8). Moreover, the expression of miR-328 was lost in blast crisis of CML and its overexpression could promote cell differentiation and inhibit cell proliferation, by inducing the survival factor PIM1 and suppressing the binding between the translational regulator poly(rC)-binding protein hnRNP E2 and CEBPA (9). Besides, miR-223 which formed a feedback loop with the cell cycle regulator gene E2F1 was found to be underexpressed in acute myeloid leukemia (AML) (10). MiR-29 was downregulated in KIT-related AML. The mutual regulation of miR-29 and SP1/NF-κB/HDAC formed a feedback regulation network, which mediated the pathogenesis of KIT-related AML (11). MiR-181 family was overexpressed in AML, which blocked cell differentiation by inhibiting the expression of PRKCD, CTDSPL and CAMKK1 (12). However, whether miR-181d dysregulation is involved in CML progression, is unknown.

The dysregulation of histone-modifying enzymes, including methyltransferases and demethylases, mediates tumorigenesis. Recent studies have found that histone methyltransferase is involved in the pathogenesis of CML blast crisis. For instance, the retinoblastoma-interacting zinc-finger protein 1 (RIZ1), a H3K9 histone methyltransferase, were downregulated in CML-BP. The overexpression of RIZ1 can inhibit cell proliferation, induce apoptosis and promote differentiation by inhibiting the IGF-1 signaling pathway (13, 14). The expression of BMI1, that belonged to the Polycomb-group of proteins, gradually increased in CML progression, which made this protein an effective marker of CML-BP (15). We have found that the histone demethylase RBP2 mediated the blast crisis of CML through a negative regulation of miR-21 (16). However, the mechanism of RBP2 underexpression in CML-BP, has not yet been studied.

The Nuclear Factor-kappa B (NF-κB) family plays an important role in CML progression by regulating cell proliferation and apoptosis and there was an NF-κB/TNF-α feedback loop, in leukemic primary cells, which promoted leukemia progression (17). The inhibitors of NF-κB signaling pathway PS-1145 and AS602868, significantly induced the apoptosis of CML primary cells (18, 19). Parthenolide, that inhibited NF-κB transcription, greatly promoted the apoptosis of leukemia cells in the blast crisis phase (20). Furthermore, recent studies showed that there was an interaction between NF-κB and histone-modifying enzymes, which were involved in the development of inflammation and tumors, such as JMJD3, FBXL11 and JMJD2B (21–23). However, whether RBP2 could regulate NF-κB expression, remains largely unknown.

In this study, we aimed to define whether miR-181d could mediate CML progression through a miR-181d/RBP2/NF-κB p65 feedback loop. In CML, miR-181d overexpression repressed RBP2 expression, which resulted in p65 overexpression. Moreover, high p65 expression reversely upregulated the level of mature miR-181d, which formed a feedback loop and promoted CML blast crisis transition.

## Methods

### Patients and Bone Marrow Samples

Patients’ bone marrow samples were collected between July 2010 and June 2018, from the Department of Hematology, Qilu Hospital of Shandong University, Jinan, China. The patients were newly diagnosed CML-CP (n=42) and CML-BP (n=15). Mononuclear cells were isolated from the samples by Ficoll-Hypaque density-gradient centrifugation and stored at -80°C. The study was approved by the Ethics Committee of Qilu Hospital of Shandong University, and also accorded with the Helsinki Declaration of 1975, as revised in 1983.

### Cell lines and Cell Culture

The human cell line K562, HL60 and HEK-293 were cultured at 37°C, 95% air and 5% CO2 in RPMI 1640 or RPMI DMEM, containing 10% heat-inactivated fetal bovine serum (FBS; Gibco, Carlsbad, CA, USA) and without antibiotics. The cells were cultured on 6,12 and 24-well plates for 18 to 24 h before the start of the experiments.

### Transfection

MiR-181d mimics (miR10002821-1-5)/inhibitor (miR20002821-1-5; Ribobio, Guangzhou, China) and p65 siRNA (SASI_Hs01_00171095; Sigma-Aldrich, USA) were transfected into the cells using Lipofectamine 2000 (Invitrogen, Carlsbad, CA, USA) and according to the manufacturer’s protocol. Plasmids, containing the RBP2 wild-type, the RBP2-mutant (defective in demethylase activity RBP2 H483A, Addgene), p65 (laboratory owned) and the control plasmid (laboratory owned), were transfected using the Roche Transfection Reagent (Roche, Switzerland).

### RNA Extraction and qRT-PCR

Total RNA was extracted from the human bone-marrow samples and the cells with different treatments using Trizol reagent (Invitrogen, Carlsbad, CA, USA). Subsequently, the extracted RNA was reverse transcribed using PrimeScript RT reagent Kit using the gDNA Eraser (Takara, Japan). The cDNAs were then subjected to SYBR Green-based real-time PCR analysis. RBP2 and p65 mRNA levels were normalized to that of the human β-actin. The level of the mature miR-181d was normalized to that of U6. The probes for RBP2 used were Hs00231908_m1 (Applied Biosystems). The primers for miR-181d and U6 were MQP-0101 and MQP-0201 (Ribobio). The other primers used in qRT-PCR assay are listed in **Table 1** and the expression was calculated by the 2^-ΔΔCt^ method.

**Table 1:** PCR Primers.

Primers		Sequences
p65	Forward	ATGTGGAGATCATTGAGCAGC
	Reverse	CCTGGTCCTGTGTAGCCATT
β-actin	Forward	AGTTGCGTTACACCCTTTCTTG
	Reverse	CACCTTCACCGTTCCAGTTTT
p65-ChIP	Forward	TGCAATGGGTACATGGGTGT
	Reverse	GTGGCTGGCCCTGATTAGAA
miR-181d-ChIP	Forward	ATGGAGTTGAGAAGGGCTGC
	Reverse	TGGGTCAGACCAGGAGAGAG

### Western Blot Analysis

Total proteins were extracted using a lysis buffer, containing protease inhibitors, and subjected to quantitative determination. The proteins were separated by SDS-PAGE gels, transferred to PVDF membranes (Millipore, Bedford, MA) and overnight probed, at 4°C, with specific primary antibodies against RBP2 (1:1000, Abcam, ab70892), p65 (1:5000, Abcam, ab32536) and β-actin (1:10000, Sigma, A5441) and followed by 1h incubation with a horseradish peroxidase-labeled goat-anti-rabbit/mouse IgG (1:6000, Abcam). Subsequently, immunoblots were probed with ECL detection reagent (Millipore) and according to standard protocols.

### Immunostaining

The mononuclear cells, that were isolated from patient bone-marrow samples, were used to prepare cytospins with glass slides fixed by a polyformaldehyde fixation solution. The samples were stained with anti-RBP2 antibody (1:150, Abcam, ab78322) and anti-p65 antibody (1:150, Abcam, ab32536) overnight at 4°C, followed by an incubation with a horseradish peroxidase-conjugated secondary antibody for 30 min.

### Immunohistochemistry

Paraffin embedded slides were deparaffinized, rehydrated and subjected to antigen-retrieval using citric acid buffer. The endogenous peroxidase was deactivated by H2O2. The slides were blocked using a 10% goat serum solution and incubated with the corresponding primary antibodies overnight at 4°C. The used antibodies were: anti-RBP2 antibody (1:150, Abcam, ab78322), anti-p65 antibody (1:150, Abcam, ab32536) and anti-Ki67 antibody (1:100, Abcam, ab15580). Next, the slides were incubated with a secondary antibody, followed by a colorimetric detection using a DAB staining kit (Vector Laboratories, USA).

### Cell Proliferation Assay

The 5-ethynyl-2′-deoxyuridine (EDU) assay was used to detect proliferative rates of K562 and HL60 cells. The treated cells were incubated with EDU for two hours before fluorescence detection. Then, the cells were smeared on glass slides, fixed with 4% paraformaldehyde for 30 minutes and then stained using a Cell-Light™ EDU Apollo^®^488 In Vitro Imaging Kit (RioBio, China), and following the manufacturer’s instructions. The slides were examined by confocal laser scanning microscopy.

### Chromatin Immunoprecipitation (ChIP) Assay

For the ChIP assay, the Cell Signaling ChIP assay protocol was used. DNA was purified from chromatin fragment immunoprecipitated with antibodies against RBP2 (Abcam, ab70892) and di- and trimethylated H3K4 (Abcam, ab32356 and ab8580), and used for PCR amplification. The precipitated DNA samples were detected by PCR. The PCR primers for p65 and miR-181d promoters are listed in **Table 1**.

### Luciferase Reporter Assay

MiR-181d mimics/inhibitor (Ribobio, Guangzhou, China), RBP2 wild-type/mutant 3′UTR and the internal control vector TK plasmids were transfected into HL60 and HEK-293 cells. The plasmids containing RBP2 wild-type, RBP2-mutant (defective in demethylase activity, RBP2 H483A), p65 promoter wild or binding site mutant plasmids, and the internal control vector TK plasmid, were transfected into HL60 and HEK-293 cells. The p65 expression plasmid/siRNA, miR-181d promoter wild, or binding site mutant plasmids, and the internal control vector TK plasmid were transfected into HL60 and HEK-293 cells. After 24 or 48 h of incubation, luciferase activity was measured using a Luciferase Assay System (Promega, Madison, WI, USA) and according to the manufacturer’s protocol. The accurate sequences of each promoter region cloned for luciferase assay are showed in **Supplemental Material**.

### Tumor Xenograft Model

For the xenograft model, 6 NOD/SCID male mice (Hua Fu Kang Biological Technology, Beijing, China) were treated with 2Gy dose of radiation. After 24 hours, 1× 10^6^ K562 cells were subcutaneously injected into the right or left flank of the mice. Tumor growth was monitored every 3 days. From the seventh day, miR-181d antagomir (miR30002821-4-5) or control, were injected into the tumor every 3 days. The total period lasted 16 days. All animal procedures were approved by Qilu Hospital of Shandong University Research Ethics Committee. The animal study also accorded with the ARRIVE guidelines (24).

### Statistical Analysis

All experiments were repeated at least three times. The data were expressed as mean ± standard deviation. Student’s t-test was used to compare the means between the two groups using the GraphPad Prism for Windows, version 5.00 (GraphPad Software, La Jolla, CA, USA). The p-values of < 0.05 were considered statistically significant.

## Results

### miR-181d Was Overexpressed in CML-BP and Promoted Leukemia Cell Proliferation

**Figure 1:**
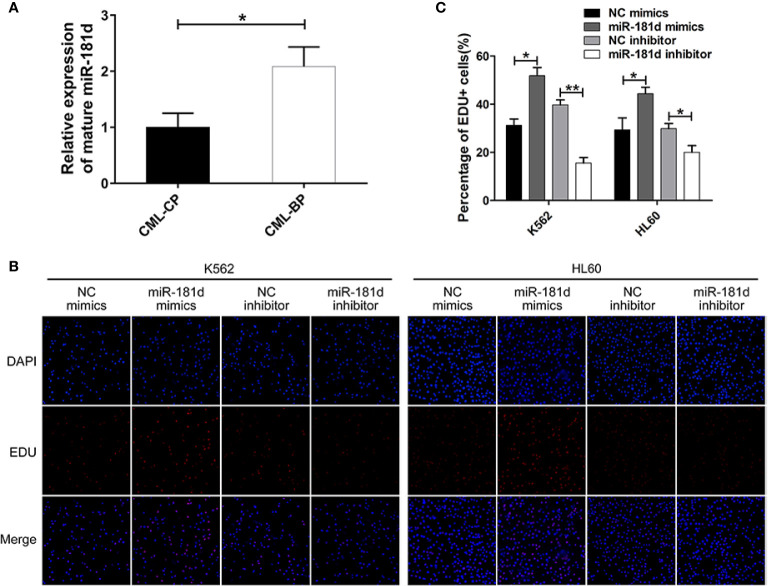
miR-181d is overexpressed in CML-BP and promotes leukemia cell proliferation. **(A)** qRT-PCR analysis of mature miR-181d in CML-CP and CML-BP patients. Human CML-CP and BP cells were obtained from patients' bone marrow. **(B, C)** EDU of K562 and HL60 cells following transfection with miR-181d mimics or inhibitor. The results were confirmed by 3 independent experiments. *p < 0.05, **p < 0.01.

To examine whether miR-181d is critical in CML progression, we measured the expression level of mature miR-181d in bone-marrow samples of newly diagnosed CML-CP patients (n=42) and CML-BP patients (n=15). The relative clinical characteristics of these patients are shown in **Table 2**. The mature miR-181d level was higher in the CML-BP samples than in the CML-CP samples (**Figure 1A**). Therefore, miR-181d was overexpressed during CML progression. Furthermore, we explored the potential role of miR-181d in leukemia cell proliferation by transfecting miR-181d mimics or inhibitor into K562 and HL60 cells. The cells transfected with miR-181d mimics proliferated at a higher rate compared to the NC mimics transfection, and the cells transfected with miR-181d inhibitor proliferated at a slower rate (**Figures 1B, C**).

**Table 2:** Patients' characteristics.

Characteristic		CML-CP patients (n = 42)	CML-BP patients (n = 15)
Gender	Male	23	9
	Female	19	6
Age(years)	Median	46	42
	Range	21-75	19-68
WBC, ×10^9^/L	Median	202.34	125.18
	Range	10.34-441.60	2.15-414.46
Hemoglobin, g/L	Median	100.38	82.8
	Range	48-147	45-136
Platelet count, ×10^9^/L	Median	469.96	59.70
	Range	134-1486	10-210

### miR-181d Directly Inhibited RBP2 Expression

**Figure 2:**
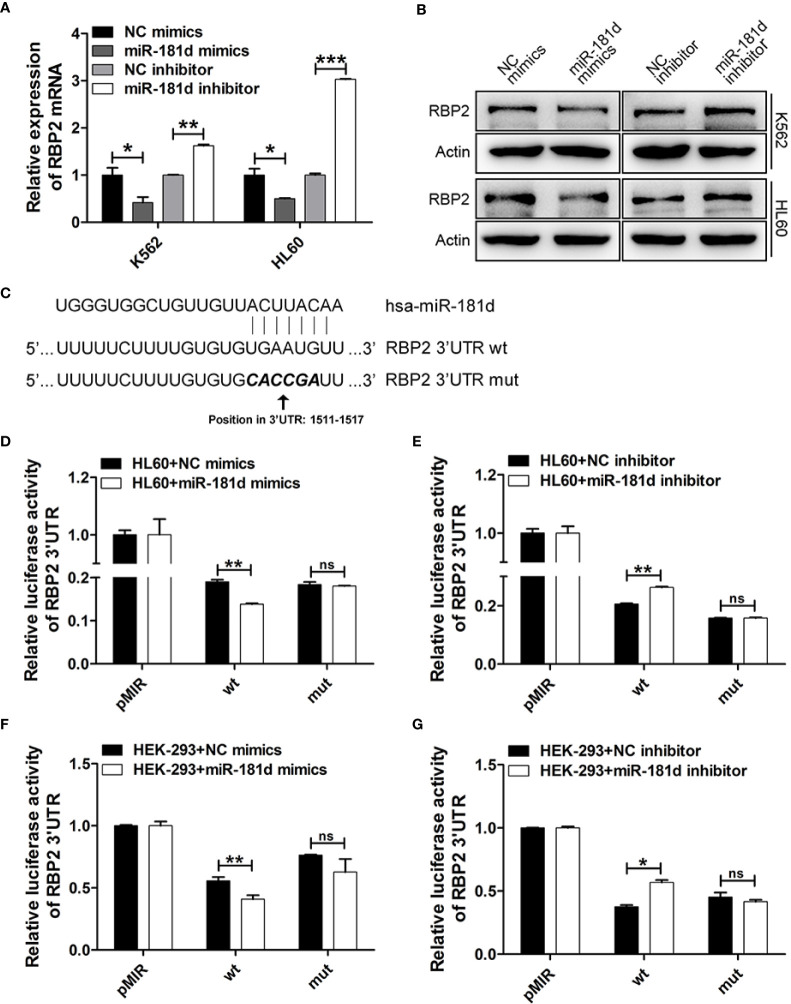
miR-181d directly regulates RBP2 expression. **(A, B)** qRT-PCR and Western blot analysis of RBP2 mRNA and protein levels after transfection with miR-181d mimics or inhibitor. **(C)** Schematic illustration of the predicted miR-181d binding sites in RBP2 3′-UTR. **(D–G)** RBP2 wild type 3′UTR and its mutated activity following miR-181d mimics/inhibitor transfection in HL60 and HEK-293 cells. Luciferase activities were determined at 48 h and normalized by Renilla luciferase activity. The results are from 3 independent experiments. *p < 0.05, **p < 0.01, ***p < 0.001, ns, not statistically significant.

To investigate the mechanisms by which miR-181d promoted cell proliferation, we investigated the potential role of miR-181d in the regulation of RBP2 expression by modulating miR-181d expression levels *via* transfecting miR-181d mimics or inhibitor into K562 and HL60 cells (**Supplemental Figure 1**). RBP2 mRNA and protein levels were significantly decreased in K562 and HL60 cells overexpressing miR-181d when compared to control cells (**Figures 2A, B**, **Supplemental Figure 2**). On the other hand, miR-181d inhibition in K562 and HL60 cells, resulted in RBP2 increased mRNA and protein levels (**Figures 2A, B**, **Supplemental Figure 2**). Using the target prediction programs miRanda and TargetScan, we found that the 3′UTR of RBP2 mRNA contained a conserved miR-181d binding site (**Figure 2C**). To verify this finding, the RBP2 3′UTR, containing the putative miR-181d binding site, and its mutant 3′UTR (with mutated miR-181d binding site) were cloned downstream of the luciferase open reading frame. These luciferase reporter constructs were co-transfected into HL60 and HEK-293 cells with miR-181d mimics or inhibitor. The luciferase activity of RBP2 3′UTR was repressed by the miR-181d mimics transfection and this repression was abrogated when miR-181d binding site was mutated. Oppositely, the miR-181d inhibitor upregulated the luciferase activity of RBP2 3′UTR and this upregulation was also abrogated when miR-181d binding site was mutated (**Figures 2D–G**). These data demonstrated that RBP2 was a direct target of miR-181d.

### RBP2 Directly Suppressed p65 Transcriptional Expression in Histone Demethylase-Dependent Manner

**Figure 3:**
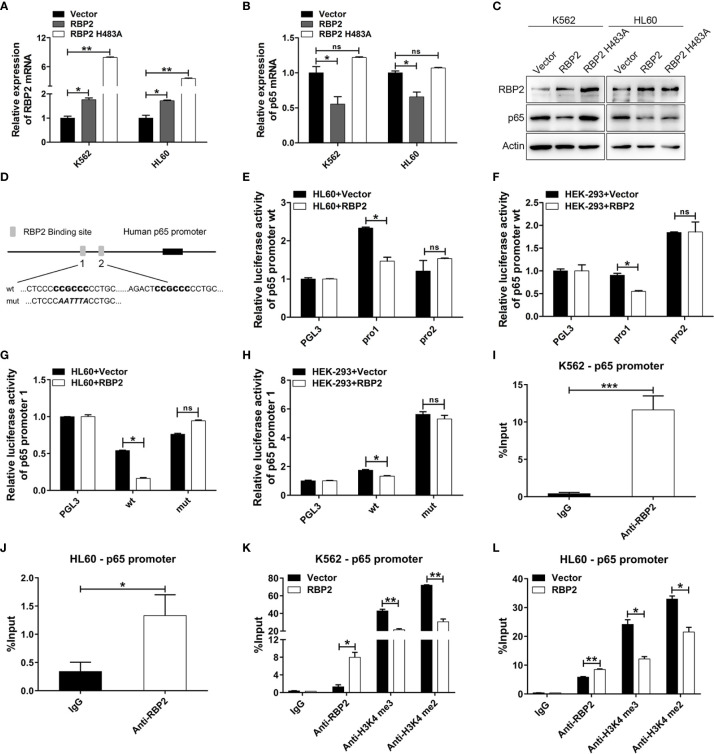
p65 is directly and epigenetically downregulated by RBP2. **(A, B)** qRT-PCR of RBP2 and p65 mRNA level after transfection with RBP2 wild-type or RBP2-mutant (defective in demethylase activity, RBP2 H483A) plasmids in K562 and HL60 cells. **(C)** Western blot analysis of RBP2 and p65 protein expression levels after transfection with RBP2 wild-type or RBP2 H483A plasmids in K562 and HL60 cells. **(D)** Schematic illustration of the predicted RBP2 binding sites in the p65 promoter. **(E, F)** Regulation of p65 wild-type promoter by RBP2, including the first RBP2 binding site (p65 pro1) and the second RBP2 binding site (p65 pro2). HL60 and HEK-293 cells were transfected with p65 wild-type reporters, together with RBP2 expression plasmids, followed by luciferase activity assessment 48 h post-transfection. **(G, H)** p65 wild-type pro1 and its mutated activity following RBP2 wild-type plasmids transfection in HL60 and HEK-293 cells. **(I, J)** ChIP assay for RBP2 binding to the p65 promoter in K562 and HL60 cells. **(K, L)** The binding of RBP2 and H3K4me3/2 to p65 promoter after RBP2 plasmid transfection in K562 and HL60 cells. The results are from 3 independent experiments. *p < 0.05, **p < 0.01, ***p < 0.001, ns, not statistically significant.

Several studies have shown that p65 is pivotal in promoting cell proliferation and our previous study confirmed that RBP2 mediates CML blast crisis. However, it is not clear whether p65 is involved in this pathogenesis. By transfecting RBP2 wild-type or RBP2-mutant (defective in demethylase activity, RBP2 H483A) plasmids or the control plasmid into K562 and HL60 cells, we compared p65 expression in differently treated cells. We found that RBP2 overexpression, significantly downregulated p65 mRNA and protein levels (**Figures 3A–C**, **Supplemental Figure 3**). However, RBP2 H483A plasmid, which also overexpresses RBP2 but without demethylase activity, could not inhibit p65 expression (**Figures 3A–C**, **Supplemental Figure 3**). These results suggest that RBP2 negatively regulates p65 expression and this was dependent on its enzyme activity.

Furthermore, to determine whether p65 is a direct target of RBP2, we identified two potential RBP2 binding sites in the promoter of p65 (**Figure 3D**). We generated two p65 promoter plasmids which included the first RBP2 binding site (p65 pro1) and the second RBP2 binding site (p65 pro2), and were co-transfected into the HL60 and HEK-293 cells with the RBP2 plasmid. The promoter activity of p65 pro1 was significantly decreased after RBP2 overexpression in HL60 and HEK-293 cells, while no significant change was observed in p65 pro2 promoter activity (**Figures 3E, F**). Moreover, accompanied by the mutation of the binding site in p65 pro1, the promoter activity of p65 pro1-mut was unchanged after RBP2 overexpression in HL60 and HEK-293 cells (**Figures 3G, H**). To determine the association between RBP2 and p65 promoter, a chromatin immunoprecipitation assay was performed, and which showed that RBP2 bound to the promoter region of the p65 promoter in K562 and HL60 cells (**Figures 3I, J**). Furthermore, RBP2 overexpression indicated its dose-dependent association with p65 promoter (**Figures 3K, L**). Consistent with its demethylase activity, which is specific for tri- and dimethylated lysine 4 on histone 3, RBP2 overexpression also remarkably reduced H3K4 tri/dimethylation at the promoter region of p65 (**Figures 3K, L**). Therefore, the results demonstrated that RBP2 reduced H3K4 tri/dimethylation by its binding to the p65 promoter region, which negatively regulated its expression.

### Ectopic Expression of p65 in CML-BP Enhanced Leukemia Cell Proliferation

**Figure 4:**
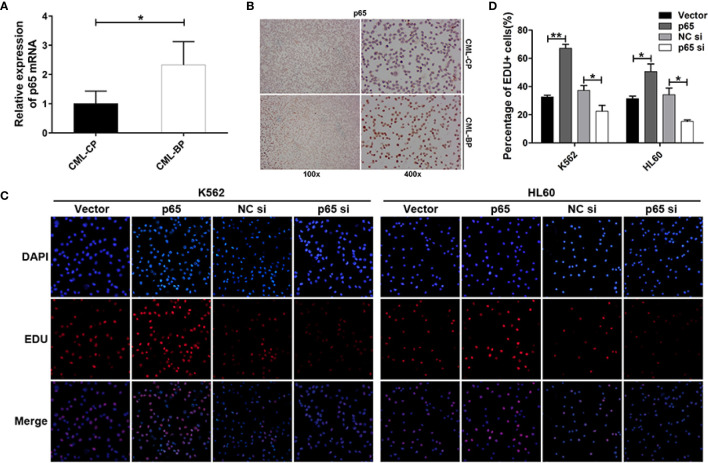
p65 is overexpressed in CML-BP and promotes leukemia cell proliferation. **(A, B)** qRT-PCR and Immunostaining analysis of p65 mRNA and protein levels in bone-marrow samples from patients with CML-CP and CML-BP. **(C, D)** EDU of K562 and HL60 cells following transfection with p65 plasmid or siRNA. The results were confirmed by 3 independent experiments. *p < 0.05, **p < 0.01.

To determine whether p65 is critical player in CML progression, we measured p65 expression in bone-marrow samples from patients with CML-CP and CML-BP. Compared with CML-CP samples, p65 mRNA and protein levels were significantly higher in the CML-BP samples (**Figures 4A, B**).

Moreover, to explore the potential role of p65 in CML progression, the p65 plasmids or siRNA were transfected into K562 and HL60 cells. The cells transfected with p65 plasmid proliferated at a higher rate compared to the control plasmid; while the cells transfected with p65 siRNA proliferated at a lower rate (**Figures 4C, D**). These data indicated that ectopic p65 expression in CML-BP cells enhanced leukemia cell proliferation.

### p65 Directly Upregulated miR-181d Expression

**Figure 5:**
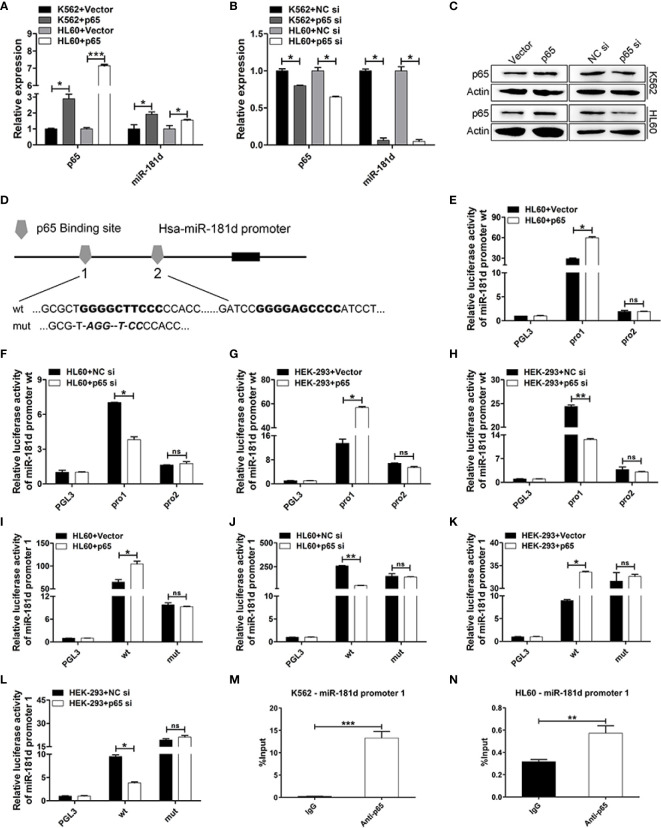
miR-181d is directly upregulated by p65. **(A, B)** qRT-PCR of p65 mRNA and mature miR-181d levels after transfection with p65 plasmid or siRNA in K562 and HL60 cells. **(C)** Western blot analysis of p65 protein expression level after transfection with p65 plasmid or siRNA in K562 and HL60 cells. **(D)** Schematic illustration of the predicted p65 binding sites in the miR-181d promoter. **(E–H)** Regulation of miR-181d wild-type promoter by p65, including the first p65 binding site (miR-181d pro1) and the second p65 binding site (miR-181d pro2). HL60 and HEK-293 cells were transfected with miR-181d wild-type reporters together with p65 plasmid/siRNA and the luciferase activity was assessed 48 h or 72h post-transfection. **(I–L)** miR-181d wild-type pro1 and its mutated form activities following with p65 plasmid or siRNA transfection in HL60 and HEK-293 cells. **(M, N)** ChIP assay for p65 binding to the miR-181d promoter in K562 and HL60 cells. The results are from 3 independent experiments. *P < 0.05, **p < 0.01, ***p < 0.001, ns, not statistically significant.

It is worth mentioning that we found miR-181d to be positively regulated by p65 (**Figures 5A–C**, **Supplemental Figure 4**). To explore this mechanism, we found that there were two classical binding sites of p65 in the miR-181d promoter (**Figure 5D**). To investigate whether miR-181d is a direct target of p65, luciferase reporters’ plasmids were constructed and which included the two potential binding sites (miR-181d pro1 and pro2), respectively. The overexpression of p65 significantly increased the luciferase activity of miR-181d pro1; while p65 siRNA greatly inhibited miR-181d pro1 luciferase activity (**Figures 5E–H**). However, the p65 plasmid and siRNA did not affect the luciferase activity of miR-181d pro2 (**Figures 5E–H**). Furthermore, we constructed a mutant luciferase reporter for miR-181d pro1 (miR-181d pro1-mut) and observed no changes in the promoter activity of miR-181d pro1-mut when p65 was overexpressed or inhibited (**Figures 5I–L**). Besides, to explore the association between p65 and the miR-181d promoter, we applied a series of ChIP assays and found that p65 bound to the promoter region of miR-181d in K562 and HL60 cells (**Figures 5M, N**). Taken together, the above results showed that p65 directly targeted miR-181d and formed a positive feedback loop to promote the transition of CML blast crisis.

### miR-181d Inhibition Suppressed Leukemia Cell Proliferation *In Vivo*

**Figure 6:**
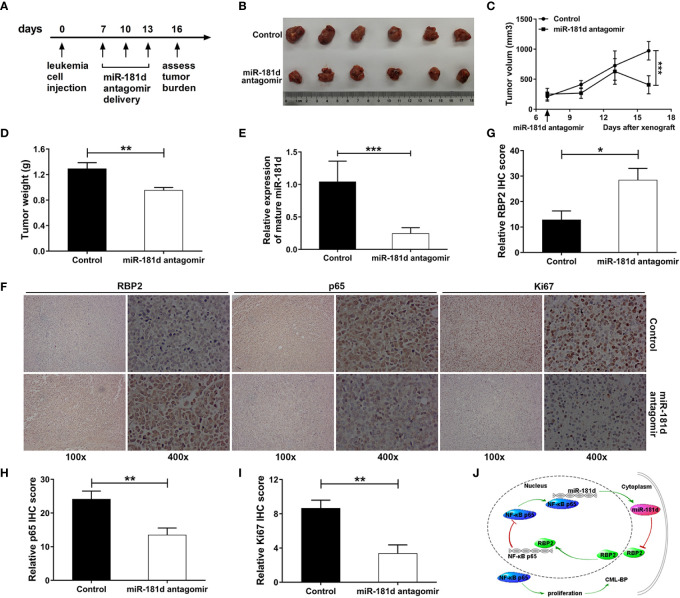
Inhibition of miR-181d suppresses leukemia cell proliferation *in vivo.* **(A)** Xenograft model in NOD/SCID male mice. Timeline of the miR-181d antagomir therapeutic delivery experiment. Primary tumor gross appearance **(B)** and tumor growth curves **(C)** and the average tumor weight **(D)** of the miR-181d antagomir or control treated xenograft model. **(E)** RT–PCR analysis shows miR-181d antagomir efficacy in each group. **(F–I)** IHC staining and relative IHC score for RBP2, p65 and Ki67 were performed on the indicated tumors. Similar results were acquired in three independent experiments. **(J)** A summary of the findings. A new epigenetic mechanism involved in the pathogenesis of CML-BP: In CML progression, miR-181d overexpression directly inhibits RBP2 expression, low levels of RBP2 cannot repress the expression of p65, which increases miR-181d expression and stimulates cell proliferation. *p < 0.05, **p < 0.01, ***p < 0.001.

We further examined the oncogenic role of miR-181d in CML blast crisis *in vivo*. When the tumor diameters reached 8 to 9 mm, miR-181d antagomir was injected into the tumor every 3 days, and then the mice were sacrificed on the sixteenth day (**Figure 6A**). The tumors, that were treated with miR-181d antagomir, were smaller compared to the control tumors treated, both in size and weight (**Figures 6B–D**). It was proved that miR-181d antagomir significantly inhibited miR-181d expression *in vivo* (**Figure 6E**). Besides, IHC confirmed that RBP2 levels were increased and that p65 levels were decreased in tumors treated with miR-181d antagomir (**Figures 6F–H**). Ki67 staining confirmed the *in vivo* tumor growth results (**Figures 6F, I**). Overall, these data indicated, that the inhibition of miR-181d suppressed leukemia cell proliferation *in vivo*, and that miR-181d might be an effective target for inhibiting CML blast transformation.

## Discussion

Recent studies have demonstrated that epigenetic dysregulation plays vital roles in leukemia. The ectopic expression of miR-181 family blocked cell differentiation by inhibiting the expression of PRKCD, CTDSPL and CAMKK1, which promoted AML pathogenesis (12). MiR-181a-1/b-1 deletion in mice, inhibited the development of T-cell acute lymphoblastic leukemia, which was induced by Notch1 (25). Except for the role of miR-181 family in leukemia pathogenesis, they also mediate drug resistance. Zimmerman EI et al. showed that the miR181 family (a–d) were underexpressed in Lyn-mediated imatinib resistant CML (MYL-R) cells. Lyn-dependent regulation of miR181 is a novel mechanism of regulating cell survival, implicating its role in drug resistance in myelogenous leukemia (26). While, drug resistance is one of the important mechanisms in CML blast crisis transition (27). The biological basis of BP is still poorly understood, and it is a complex molecular network mechanism that drives the disease progression. Among which, whether miR-181d plays important roles have not been clearly identified.

In this study, we demonstrated that miR-181d mediates CML malignant transformation and that it is overexpressed in bone-marrow samples of CML-BP. Following this, we determined that miR-181d overexpression significantly promoted leukemia cell proliferation. The possible mechanism by which miR-181d regulates cell proliferation was reported to be associated with the regulation of NKAIN2, CDKN3 and CYLD (28–30). As the targets of miR-181d, NKAIN2 knockdown could reverse the inhibition of miR-181d downregulation on pancreatic cancer development (28), CDKN3 mediated the pathogenesis of non-small-cell lung cancer (NSCLC) (29), while CYLD regulated invasion-mediated epithelial-mesenchymal transition (EMT), which resulted in gastric cancer (30). Besides, CYLD acted as a crucial regulator of Adult T-cell leukemia/lymphoma (ATLL) survival (31). The increasing studies suggest that these reported targets play important roles in tumorigenesis, including leukemia. This is also illustrated the pivotal role of miR-181d by the side. Here, focus on epigenetic dysregulation, we revealed a new mechanism and showed that miR-181d promotes leukemia cell proliferation by directly targeting and negatively regulating the histone demethylase RBP2.

The retinoblastoma binding protein 2 (RBP2) is a member of the JARID family of proteins, which has a histone demethylase activity by specifically demethylating tri- and di-methylated lysine 4 of histone 3 (H3K4) (32). RBP2 mediated the pathogenesis of a variety of tumors, such as small cell lung cancers (SCLCs) (33), gastric cancer (34), Renal cell carcinoma (RCC) (35), et al. Except for solid tumors, in hematologic malignancy, our previous study showed that a low RBP2 expression could not repress miR-21 expression, which promoted the transition of CML from CP to BP (16). Furthermore, the under-expression of RBP2 promotes CML progression by activating an RBP2/PTEN/BCR-ABL cascade (36). These studies show that RBP2 expression imbalance is an important initial factor of tumor development. However, the reason for RBP2 expression imbalance in CML blast crisis is still unknown. In this study, the results characterized the mechanism by which RBP2 was underexpressed in CML blast crisis, which further revealed the importance of epigenetic dysregulation in CML blast transformation.

The AT-rich interaction domain of RBP2 can recognize a specific DNA sequence CCGCCC (37) that is contained in the promoter region of p65. We found that RBP2 directly and negatively regulates p65 expression by binding to its promoter, which depended on its enzyme activity. RBP2 overexpression significantly downregulated p65 mRNA and protein levels. However, RBP2 H483A plasmid, which is also overexpress RBP2, but without demethylase activity, could not inhibit p65 expression. Furthermore, Luciferase reporter assay and ChIP assay showed that RBP2 directly binds to p65 promoter.

Recent studies have shown that miRNAs play important roles in inflammation and cancer by regulating the NF-κB pathway (38, 39). Nevertheless, whether miRNAs could be reversely regulated by NF-κB is largely unknown. Here, we found that NF-κB/p65 upregulates the expression of miR-181d and forms a feedback loop. Mechanistically, miR-181d proximal promoter contains the classical binding sites of NF-κB/p65 (GGGRNNYYCC R-purine, Y- pyrimidine, N- arbitrary base) (40). The overexpression of p65 significantly upregulated miR-181d expression and activated its promoter activity; while p65 inhibition greatly downregulated miR-181d expression and suppressed miR-181d promoter activity. However, when the binding site was mutated, the corresponding activation, or inhibition disappeared. Furthermore, the ChIP assay showed that p65 directly binds to the promoter sequence of miR-181d.

In summary, we provided evidence for the existence of a new epigenetic mechanism that was involved in CML blast transformation (**Figure 6J**). The non-coding RNA miR-181d was overexpressed in CML-BP, which promoted leukemia cell proliferation. Mechanistically, miR-181d downregulated the level of histone demethylase RBP2, which inhibited p65 expression in leukemia cells by its binding to the p65 promoter and demethylating the tri/dimethylated H3K4 region in the p65 promoter locus. Conversely, p65 upregulated the level of mature miR-181d by directly binding to its promoter. These findings might point to a way to build new diagnostic markers for CML blast crisis.

## Data Availability Statement

The original contributions presented in the study are included in the article/**Supplementary Material**. Further inquiries can be directed to the corresponding author.

## Ethics Statement

The studies involving human participants were reviewed and approved by Ethics Committee of Qilu Hospital of Shandong University. The patients/participants provided their written informed consent to participate in this study. The animal study was reviewed and approved by Ethics Committee of Qilu Hospital of Shandong University.

## Author Contributions

CC contributed to study design, the discussion of the results, and revised the manuscript. MZ contributed to data collection, analysis, and interpretation. XY contributed to validation of internal regulatory mechanisms. LZ and YW contributed to statistical analysis and tumor xenograft model. YF, XW, TH, and JJ contributed to the discussion of the results. ZC and ZG collected the patients’ bone marrow samples. All authors contributed to the article and approved the submitted version.

## Funding

We thank the grant support from the National Natural Science Foundation of China (grant nos. 81670146, 81470318, 81772151, 81971901, 82070164), the Key Research and Development Project of Shandong Province (grant no. 2017GSF18109), Natural Science Fund of Shandong Province (grant nos. ZR2018PH013, ZR2019PH073), Department of Science and Technology of Shandong Province (grant no. 2018CXGC1208), Science Foundation of Qilu Hospital of Shandong University (grant no. 2016QLQN09, 2017QLQN34), and the Shandong Provincial Key Laboratory of Immunohematology Open Research Program (grant no. 2019XYKF006).

## Conflict of Interest

The authors declare that the research was conducted in the absence of any commercial or financial relationships that could be construed as a potential conflict of interest.
