# Supplementary material for: miR-181d/RBP2/NF-κB p65 Feedback Regulation Promotes Chronic Myeloid Leukemia Blast Crisis
Source: Front Oncol. 2021 Mar 25;11:654411. doi: 10.3389/fonc.2021.654411 (PMC8027495; doi:10.3389/fonc.2021.654411)
Supplement: Supplementary file 1 [file DataSheet_1.pdf]

**Supplemental method:**

The accurate sequences of each promoter region cloned for luciferase assay are showed as follows:

RBP2 3’UTR:

CTGTCTGTTGGGAAACTTGGTCTCACTCTAGTCTGTGCTGGCTCTTTTTTCT
TTTGTGTGTGAATGTTTGAAACTATGGTGCTGTGTCCTCTTCCCTCCTGTTG
TGTTCTCTGTTCTTCTTAAGGTATTAGGTAAGCTGTATGTAAACTCTTATTAG
AAGGGGACTAATTTTTTTTTAAAGGACAAAAAAAAAAAATGACTAAAAATAG
TGTTTTGTCATCACTGTCTGGTGTCTTTATTTTGGTTTAAAAAAATCTGTGGT
TTGACTTAAATTCATCAGTTTTCCTTTTAAAGGGGGGATTGGGGTGGACCTA
GCAACAGCTTATAGTTTCCTCTTCCCTTTTTGCCCCAGTCTCTTTTAAAGAA
CTGTCTTCTAGTAACTAGAAGTGAAATGTACTGTCCAGTTACAGTTTGAGTG
GGTATGAGATTTAACTCAAAAGGAATCTTACAAAAAAAAAAGATGTTTTCTT
ATAAAATCCAATTTCTGTAAATGTTTTCTCTGAAGTTCATACTACCTACTTTT
TATTCATTCTTAATACTGTATAACATTTTGAGTGTTTTGACTTGTTCAGAGGT
TTGTTAGTGTTGCTGTGCATATG

The p65 promoter1:

tacagaattctggatggtggtggccccttgagttttgcaatgggtacatgggtgtttatcgtattaatttaaaagttataaatatac
atgaattttaagaagaggatccatgcaaggttcaatgataattgtatatcattaaccaaagattttaattaatccattctgtcacct
gaagtcggcctcccccgcccccctgccaaaagtaaacacttttctaatcagggccagccactggagaaactgaaatgagtg
ggcggggtgct.

The p65 promoter2:

gtttcttcagagcctgggaaacaagtcagaatttccaatcttttccaattcccacatgatttcctaatttaagacgaattcagaga
aaataaaaaagtctttctccatctggagaagactccgccccctgcagtggagcatcctcctttggggatgaggcctggggtg
ggggtgcgtcagccccagcctccacacggacgcgccttgggcttggtcttgggggagctacctgcactgtggggtcacat
gacagaattagtggtcagccctagctggaggtccagggcaaacacaggcgggggcagggggggtggggggtcgtgagc
ctgctgattcaagtgtccctgtgggtcccgca

The miR-181d promoter1:

tttctctcggtcgatgcctgagtgtgcaaactacacccacatctgagtatacaggggcacctccgaagacccccacggcct
gacacatccacccagtgtccaatggccacagccttgggggacttcgggggctggagttccgggctgatggagttgagaa
gggctgcctggctcttacacccaccttgggtccccacggagacccccaaggggacaggaggtgaaatctcccagggag
aggttcaggaagagctgctgctgtgtcgcagccctgggctcccccctatgagtcagcactggcaggggccccaggaactg
gggaccgggggtgtgtagggggggagggcagttcccacgccctaaggcgtctggggcgctggggcttcccccaccgctc
ctcccatcctgtgctgggctctggggatcttcagaggagtctggggggtgagaagcccagggcggattggccactgggc
caggatccaggaatcctctctcctggtctgacccagttcaggaatgcagaggccgcccgccccggcaaatgcgttgcagc
aagaaggagagttagacgtagagaaacaccgagtgagttggacatttgtgtggacggcacttctaggggggctttatttaaga
aggggcccttccttctggggactctgcatttgagcagtggaggctccagcctggggtgtccaggcagagtcctcagctacc
gtggagtgggaggtggcggggaggaggggggaggagactgcgctgagagctgagatcagtcccgccggcctccacag
ctgggagggcgctggactctgctgccccctgctggtggctgcaggaaattcagcgagcaggacagctggccg

The miR-181d promoter2:

tggaggaggctgaaaggaagtgaggggtgcgcgatcaggtggggaagggccatggccccataagggttaaggtctgg
gcttggaagggggccagtcaaaggtttctccatccccctctcagactggggtgggggataaataaacagaatgagaaatct
gtcagctcagcccgggccaatgggaggctgcctggcgccatggtttgccctgatgtgaccttcctctaccagccgcggtg
gccactgcgggcaggggcagtgtgtggagcctcagccagggtacgatcattccagccttgtgcatgctcttatacctgggc

agggggagggggggatgtctggggaggagtaccgtctgtgcaaggatctttgggagatccggggagccccatcctttgtac agcttgcatgtggtctgtgcacctggcctgtatatatgcttgtgcctcatggtcccaggtacacatccccacgtgcccctgaat tgcgtgtgtccatgtggacccctgtggctctgggacctgctgggggtcttgtgcatgtggctgttggctgtgtttctctgcctct gcgactggtccatttgacc

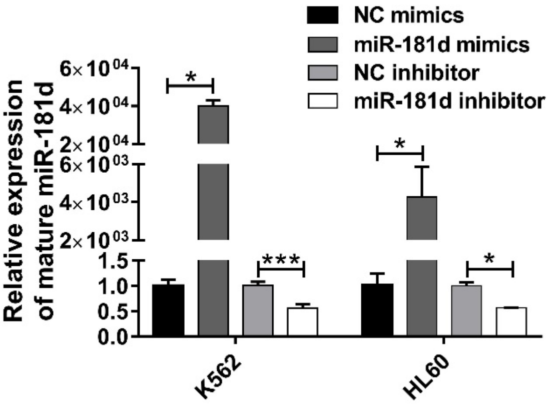


**Supplemental Figure 1. The expression of miR-181d in K562 and HL60 transfected cells.**

qRT-PCR analysis of mature miR-181d levels after transfection with miR-181d mimics or inhibitor in K562 and HL60 cells.

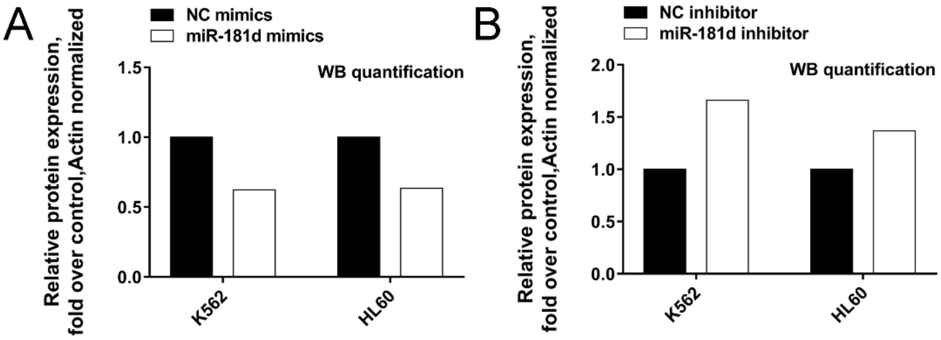


**Supplemental Figure 2. Relative expression of RBP2 protein level in K562 and HL60 transfected cells.**

(A, B) Relative protein levels of RBP2 were quantified using the ImageJ program, with Actin for normalization.

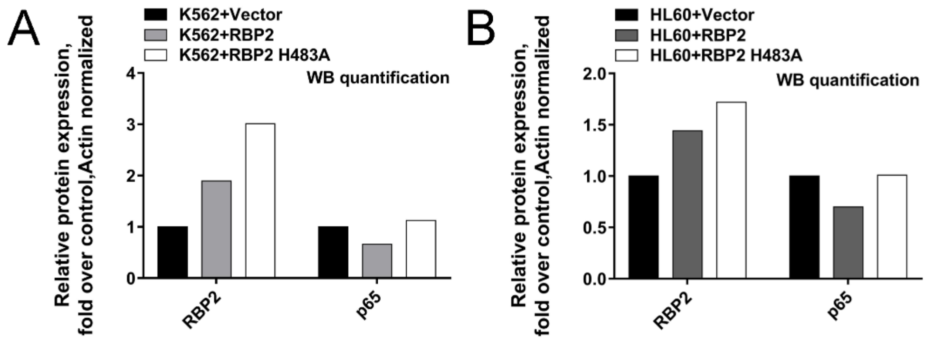


**Supplemental Figure 3. Relative expression of RBP2, p65 protein level in K562 and HL60 transfected cells.**

(A, B) Relative protein levels of RBP2 and p65 were quantified using the ImageJ program, with Actin for normalization.

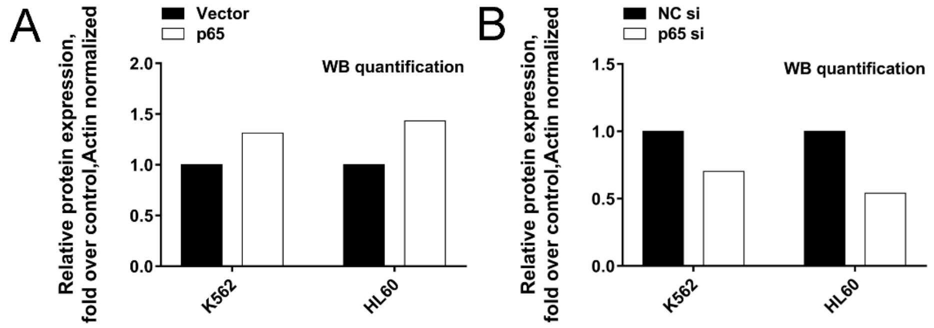


**Supplemental Figure 4. Relative expression of p65 protein level in K562 and HL60 transfected cells.**

(A, B) Relative protein levels of p65 were quantified using the ImageJ program, with Actin for normalization.
